# PW02-030 - Clinical phenotype in individuals with Q703K

**DOI:** 10.1186/1546-0096-11-S1-A171

**Published:** 2013-11-08

**Authors:** DM Rowczenio, H Trojer, G Wang, PN Hawkins, HJ Lachmann, A Baginska, T Russell, R Al-Nackkash, A Bybee, NM Stewart, T Lane

**Affiliations:** 1 Medicine, University College London, London, UK

## Introduction

Mutations in the *NLRP3* gene are associated with the dominantly inherited cryopyrin-associated periodic syndrome (CAPS) characterized by episodes of fever, urticarial rash, arthralgia, myalgia, eye inflammation, and, in its more severe forms, bony abnormalities and CNS inflammation. Of the 145 sequence variants in *NLRP3* reported to date, 30 are either nonpathogenic or of undetermined significance, the commonest of which, Q703K, has been reported in 5 to10% of general population.

## Objectives

To characterize the clinical phenotype in individuals with Q703K in a single UK center.

## Methods

1017 subjects with hereditary periodic fever syndromes (HPFS) were screened for mutations in the *NLRP3* gene, individuals in whom genetic variants were not identified or those with low penetrance mutations underwent additional screening of *MEFV*; *TNFRSF1A*; *MVK* and *NOD2*.

## Results

*NLRP3* Q703K was identified in 69 subjects (7% of screened), clinical information was available on 56. 4 cases had another mutation in *NLRP3*: 1 had A439V and 3 siblings had R260W. 18 subjects (32%) had aberration in another HPFS gene: 4 in *TNFRSF1A*: R92Q, C29F, H22Q and S57_E64del; 1 in *MVK*: V377I; 2 in *NOD2*: P268S and 12 in *MEFV*: 2 were compound heterozygotes, M680I/V726A and M694V/V726A, 2 were homozygous and 1 was heterozygous for M694V, 1 was heterozygous for V726A, 2 for S208C, 1 for S154P and 3 for E148Q.

The inflammatory syndromes were thought to be fully consistent with CAPS, TRAPS, MKD and FMF in the 4 cases with *NLRP3* variants other than Q703K; 4 subjects with *TNFRSF1A* mutations; a subject with *MVK* variant and 5 of the 12 cases with *MEFV* substitutions respectively. One subject with Q703K and E148Q was an asymptomatic carrier and in 4 cases a diagnosis of disease other than HPFS was made. 14 cases (25%) were diagnosed with AA amyloidosis (confirmed immunohistochemically and by SAP scintigraphy) the nature of the underlying inflammatory disease in 12 remains uncertain. In total we were unable to make a clinical diagnosis in 25 subjects (44%): in this group the median age at disease onset was 5 years (birth-59 years); fever, arthralgia and myalgia were the most prominent features - identified in over 50% of cases; 11 subjects (44%) had rash during febrile attacks (urticarial rash was reported in 4); 7 (28%) had symptoms triggered or worsened by cold exposure; 5 (20%) suffered from headache, GI symptoms or lymphadenopathy; 4 (16%) had hearing impairment; a delayed puberty was identified in 4 (16%) and one had growth deficit. Episodes occurred irregularly and lasted from 1 day to 2 weeks.

In 10 the inflammatory markers, serum amyloid A protein (SAA) and C-reactive protein (CRP), were measured during disease flare and were elevated to median values of 106.5 mg/L (range 40-438) and 68 mg/L (range 34 – 220) respectively.

## Conclusion

We have identified Q703K in subjects displaying FCAS-like symptoms, in individuals with HPFS other than CAPS, in cases with uncharacterised autoinflammatory diseases, in AA amyloidosis, and in asymptomatic individuals. Given the high frequency of healthy carriers, the interpretation of Q703K presents a diagnostic challenge and the genetic and/or environmental factors that may influence pathogenic consequences of this variant remain unknown.

## Competing interests

None declared

